# *PyCycleBio*: modelling non-sinusoidal-oscillator systems in temporal biology

**DOI:** 10.1093/bioadv/vbag018

**Published:** 2026-01-22

**Authors:** Alexander R Bennett, George Birchenough, Daniel Bojar

**Affiliations:** Department of Medical Biochemistry, Institute of Biomedicine, University of Gothenburg, 41390 Gothenburg, Sweden; Department of Medical Biochemistry, Institute of Biomedicine, University of Gothenburg, 41390 Gothenburg, Sweden; Wallenberg Centre for Molecular and Translational Medicine, University of Gothenburg, 41390 Gothenburg, Sweden; Wallenberg Centre for Molecular and Translational Medicine, University of Gothenburg, 41390 Gothenburg, Sweden; Department of Chemistry and Molecular Biology, Institute of Biomedicine, University of Gothenburg, 41390 Gothenburg, Sweden

## Abstract

**Motivation:**

Protein, mRNA, and metabolite abundances can exhibit rhythmic dynamics, such as during the day–night cycle. Leading bioinformatics platforms for identifying biological rhythms often utilize single-component models of the harmonic oscillator equation, or multi-component models based upon the Cosinor framework. These approaches offer distinct advantages: modelling either temporally resolved regulatory behaviour via the extended harmonic oscillator equation, or complex rhythmic patterns in the case of Cosinor.

**Results:**

Here, we have developed a new platform to combine the advantages of these two approaches. *PyCycleBio* utilizes bounded-multi-component models and modulus operators alongside the harmonic oscillator equation, to model a diverse and interpretable array of rhythmic behaviours, including the regulation of temporal dynamics via amplitude coefficients. We demonstrate increased sensitivity and functionality of *PyCycleBio* compared to other analytical frameworks, and uncover new relationships between data modalities or sampling conditions with the qualities of rhythmic behaviours from biological datasets—including transcriptomics, proteomics, and metabolomics. We envision that this new approach for disentangling complicated temporal regulation of biomolecules will advance chronobiology and our understanding of physiology.

**Availability and implementation:**

*PyCycleBio* is available at: https://github.com/Glycocalex/PyCycleBio, and the Python package is available to install at: https://pypi.org/project/pycyclebio/. *PyCycleBio* can also be used at https://colab.research.google.com/github/Glycocalex/PyCycleBio/blob/main/PyCycleBio.ipynb with no installations necessary.

## 1 Introduction

Behavioural and physiological processes often exhibit rhythmic dynamics, which are adaptations to regular phenomena such as the day–night cycle. Many chronobiological phenomena can be accurately described with a sinusoidal curve—the simplest model providing stable oscillatory behaviour—and sinusoidal oscillations are commonly used to identify rhythmic biological molecules. However, it has long been noted that many rhythmic processes violate the sinusoidal assumption. For example: theoretical work has demonstrated that non-sinusoidal oscillations are required for circadian temperature compensation; behavioural data exhibits non-sinusoidal rhythms in many cases; and many other biological rhythms exhibit non-sinusoidal dynamics, such as sexual or seasonal cycles. These biological rhythms remain relatively underexplored, and improved bioinformatics support for complex-rhythm analysis may facilitate future studies in these areas. Since current methods usually cannot easily model these phenomena, we currently have an incomplete understanding of how biological rhythms manifest, which is partly due to a lack of non-sinusoidal modelling techniques.

Since its introduction to the field, the Jonckheere-Terpstra-Kendall (JTK) algorithm (Hughes *et al*. 2010) has been widely adopted as an effective and approachable framework for describing sinusoidal expression patterns, using a non-parametric mapping of the harmonic oscillator equation. More recently, De Los Santos *et al*. (2020) have expanded upon the framework of ‘JTK’ by parametrically modelling the extended harmonic oscillator equation and patterns of expression exhibiting linear increases or decreases in amplitude over the temporal period of analysis. Additionally, the ‘Cosinor’ model has long been used for biological rhythm analysis, and has been developed into a Python package (Moškon 2020). Cosinor utilizes a multi-oscillator system for parameterizing temporal data and, critically, modern Cosinor analysis can model non-sinusoidal rhythms. Thus, both Cosinor and ECHO facilitate the understanding of more complex regulatory mechanisms, and they represent the current gold-standard for statistical analysis of biorhythmic data. Presently however, the advantages offered by both of these platforms have remained largely distinct. The harmonic oscillator used in ECHO analysis is unable to model non-sinusoidal temporal behaviours arising from a multi-component system. Resulting in many genuine biological rhythms being misclassified as non-rhythmic or incorrectly parameterized with sinusoidal models. Cosinor frameworks are commonly unable to model temporarily resolved regulatory dynamics, though leading platforms such as CosinorPy, GLMMconsinor and RhythmCount include this functionality. Nevertheless, though Cosinor frameworks can parameterize complex waveforms, relating these signals to the regulatory elements giving rise to observed oscillations is challenging with unbounded relationships between composite signals. For these reasons, we have developed *PyCycleBio*, an analytical framework that couples the modulatory coefficient of the extended harmonic oscillator equation with bounded-multi-component waveforms, inspired by Cosinor. Through *PyCycleBio*, we combine the best elements of both approaches into a functional and easy-to-use toolset which models sinusoidal and complex oscillations in a data-driven manner. Additionally, by utilizing logical and modulo functions we are able to capture impulse dynamics. Overall, we present *PyCycleBio* as a new foundation for data-driven chronobiology.

## 2 Methods

*PyCycleBio* is capable of modelling: (i) dampened or forced sine waves, using the extended harmonic oscillator equation, (ii) ‘pseudo-square waves’, approximating the digital waveform with two sinusoidal components, (iii) ‘cycloid waves’, where sinusoidal expression is modulated by a second sine wave of double amplitude but half-periodicity, and (iv) ‘transients’, where a modulo operator is used to produce periodic increases in magnitude from a stable baseline. The extended harmonic oscillator equation is used in the state-of-the-art bioinformatics platform ECHO (De Los Santos *et al*. 2020), and we have utilized the same equation for the parameterization of sinusoidal oscillations in *PyCycleBio:*


(1) $$x(t)=Ae^{\frac{\gamma t}{2}}\;\text{cos}(\omega t+\phi )+y$$

Where x(t) is the amplitude at time *t*, A is the initial amplitude (*t *= 0), γ is the amplitude change coefficient, ω is the frequency of oscillation, ɸ is the phase shift, and y is the equilibrium value.

We have expanded upon (1) with a pair of equations that each feature two sinusoidal terms: The ‘pseudo-square wave’ equation approximates the digital square waveform using two oscillators as follows:


(2) $$x(t)=Ae^{\frac{\gamma t}{2}}\;\text{sin}(\omega t+\phi )+0.25\;\text{sin}(3\omega t+3\phi )+y$$

**Figure 1:**
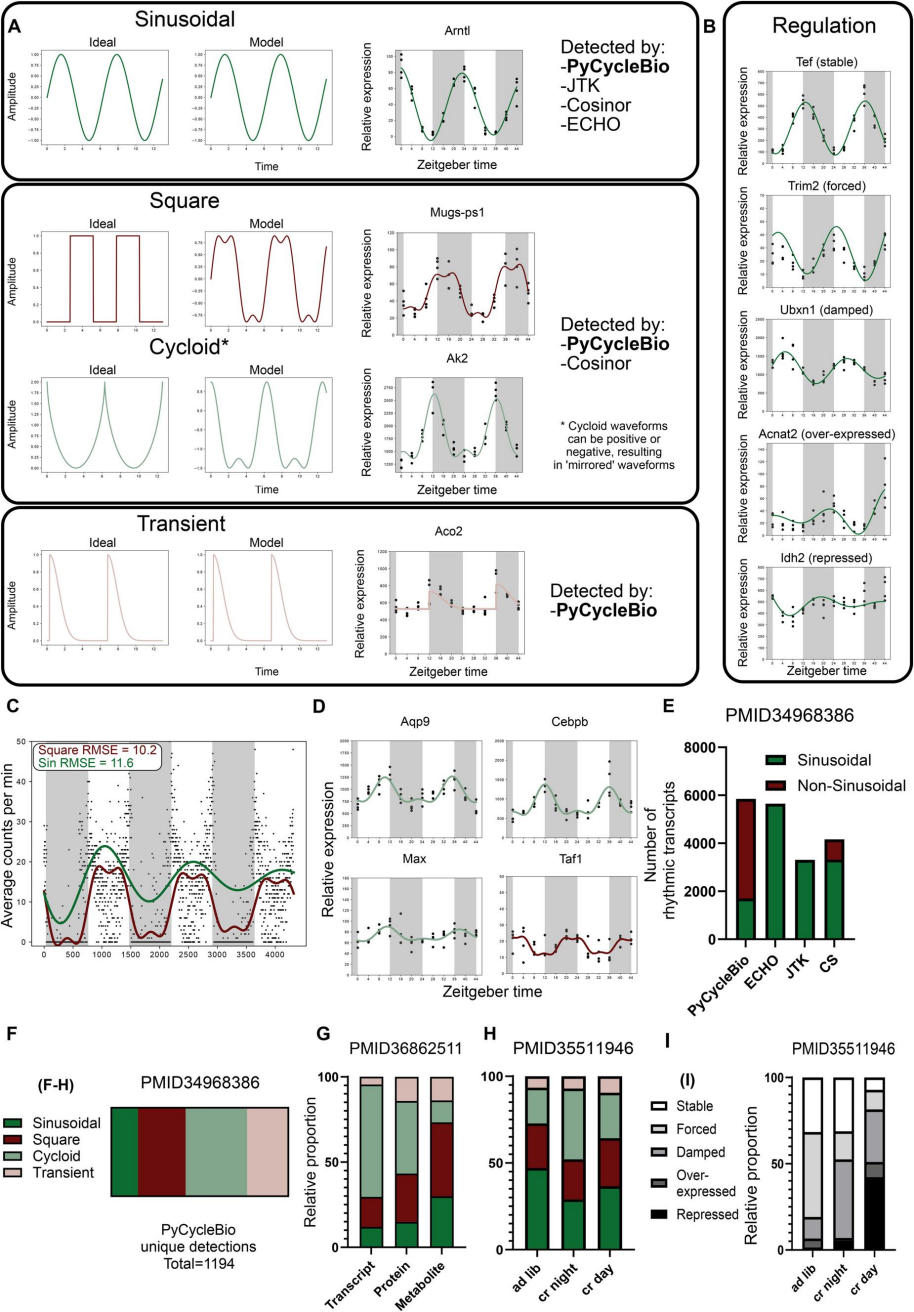
Waveform characterization, model performance, and interpretation. (A) Plots visualizing an ideal waveform, our modelled representation, and an example transcript corresponding to each type of waveform (Aviram *et al*. 2021). (B) Examples of regulatory classifications based upon γ in (1–3) (Aviram *et al*. 2021). Transcriptomics data are presented as mean ± SD of *z*-score normalized expression data. (C) An example of the optimal square and sinusoidal models produced to fit behavioural data (data adapted from Cichewicz and Hirsh 2018). (D) Expression of *Aqp9* and transcription factors *Cebpb*, *Max*, and *Taf1* (Aviram *et al*. 2021). (E) The total number of rhythmic transcripts in a transcriptomics dataset (Bignon *et al*. 2023), as identified by *PyCycleBio* and other common analytical platforms. (F) Distribution of assigned models in transcripts (Bignon *et al*. 2023) identified as rhythmic by *PyCycleBio* and not by *ECHO.* (G) Distribution of models in three tissue-matched ‘omics modalities, generated from murine renal tissue (Bignon *et al*. 2023). (H) Distributions of models and (H) regulatory behaviour of oscillating molecules, within the hepatic transcriptome of mice fed *ad-libitum*, for two hours during the night or for two hours during the day (Acosta-Rodríguez *et al*. 2022).

In (2), the carrier waveform is modulated using the sum of a second sinusoidal oscillator with one-third the periodicity and half the amplitude of the file:///C:/Users/Alex%20Bennett/Downloads/PyCycleBio_fig_1.pdf carrier.

The ‘pseudo-cycloid wave’ is another revision of (1), and uses two cosine components to approximate the dynamics of a cycloid wave:


(3) $$x(t)\;=\;Ae^{\frac{\gamma t}{2}}*-0.5(\text{cos}(2\omega t+2\phi )-2\;\text{cos}(\omega t+\phi ))+y\;$$

Finally, we have utilized a modulator to apply a Gaussian function at specific intervals, producing transient expression impulses from a stable baseline, where τ represents 2*π, and p_τ_ represent impulse periodicity:


(4) $$x(t)=A*\left\{\begin{array}{cc} e\left(-\frac{1}{2}{\left(\frac{t\;\text{mod}\;\tau -p_{\tau }}{\omega }\right)}^{2}\right) & \text{if}\;t\;\text{mod}\;\tau -p_{\tau }\geq 0\\ 0 & \text{otherwise} \end{array}\right\}+y$$

### 2.2 Model selection

*PyCycleBio* uses the *optimize.curve_fit* function from SciPy (version 1.11.1) to produce an optimal parameterization for each model via non-linear least square. These parameters are then used to produce fitted values, and the residuals between fitted and experimentally measured values are computed. The model with the lowest sum of squared residuals is determined to be optimal. Root mean squared error (RMSE) is included in user outputs alongside model parameters, indicating the quality of model fit. Following optimal model selection a lack-of-fit F-statistical test is performed to filter signals with low variance or high noise, between the observed measurements and the best model, with all optimal parameters but zero-amplitude. Molecules that exhibit significant lack-of-fit with the zero-amplitude model are deemed rhythmic. This mitigates the detection of false-positives during our analyses, especially from low-variance molecules. Finally, Kendall’s tau algorithm is used, from the implementation available in the *stats* module of SciPy (version 1.11.1; Virtanen *et al*. 2020), statistically determining how accurately fitted values represent experimental values. Model outputs, including equation parameters, RMSE, categorized regulatory behaviour, and modelled values, are provided alongside the *P*-value of the optimal model. We additionally report the Benjamini-Hochberg corrected *P*-values.

## 3 Results

### 3.1 PyCycleBio models distinct rhythmic waveforms

*PyCycleBio* is able to parameterize: sinusoidal, cycloid, square, and transient patterns of expression, with amplitude coefficients to indicate active regulatory processes. Idealized versions of these waveforms, as well as example transcriptomics data (Bignon *et al*. 2023) exhibiting these forms of rhythmic expression are visualized in Fig. 1A, with examples of regulatory classifications in Fig. 1B. We note that behavioural data in particular is poorly modelled with sinusoidal waves. Locomotor activity data from *Drosophila melanogaster* (Cichewicz and Hirsh 2018), recorded by the *Drosophila* Activity Monitor system (Cichewicz and Hirsh 2018), was analysed with *PyCycleBio* and confirmed a square waveform to be more suited for parameterization than a sine wave, corroborated by a lower RMSE (Fig. 1C).

Transcriptomics data from murine liver tissue (Aviram *et al*. 2021) demonstrates how *PyCycleBio* can facilitate interpretation of the regulatory insights giving rise to observed regulatory dynamics. For example, the cycloid dynamics observed in *Aqp9* expression (Fig. 1D) are modelled by (3), which describes the additive effects of two oscillatory elements: one of which is circadian and the other ultradian, with a period of ∼12 hours. This is corroborated by the observed expression of transcription factors *Cebpb*, *Max*, and *Taf1*. *Aqp9* has two promoter regions: *Cebpb* binds one promoter and has a 24-hour rhythm at the transcript level; the second *Aqp9* promoter is bound by both *Max* and *Taf1*, which oscillate with anti-phase 24-hour rhythms, suggesting that one of these factors is active on the promoter every 12 hours. Together, the dynamics of these three transcription factors activating two promoter regions could explain the observed expression dynamics of *Aqp9*. We also note that *PyCycleBio* offers substantially improved performance (Fig. 1A, available as supplementary data at *Bioinformatics Advances* online) and greater accuracy (Fig. 1B, available as supplementary data at *Bioinformatics Advances* online) than other common platforms with synthetic (Ness-Cohn *et al*. 2020) and biological (Benegiamo *et al*. 2018, Aviram *et al*. 2021, Acosta-Rodríguez *et al*. 2022, Bignon *et al*. 2023) data.

### 3.2 The majority of unique rhythms detected by PyCycleBio are non-sinusoidal

We find *PyCycleBio* to be more sensitive in detecting oscillatory behaviours than other commonly used bioinformatics packages (Fig. 1E): in a previously published dataset 4652/15 576 rhythmic molecules were detected by both *PyCycleBio* and ECHO, 1194/15 576 by *PyCycleBio* alone, and only 995/15 576 by ECHO alone (Fig. 1C, available as supplementary data at *Bioinformatics Advances* online) (Bignon *et al*. 2023). Additionally, the RMSE of *PyCycleBio* (7.89) compared to ECHO (9.13) when modelling *Arntl* (sinusoidally in both cases) suggests more accurate modelling (Aviram *et al*. 2021). Although increased detection is an attractive selling-point for analytical algorithms, minimizing false-positives is essential too. *PyCycleBio* thus uses a false-discovery-control procedure using the Benjamini-Hochberg correction. Additionally, we note that, although the total number of rhythmic molecules was increased in a *PyCycleBio* analysis compared to other frameworks, the number of sinusoidal oscillations was reduced, suggesting many rhythmic molecules were better classified using our non-sinusoidal models. Reanalyzing the dataset generated by Manella *et al*. (2021) with *PyCycleBio’*s ‘get_pycycle’ function revealed that only 15.1% of the oscillatory molecules detected by *PyCycleBio*, but not ECHO, were best modelled using the harmonic oscillator equation (Fig. 1F). This indicated that, although *PyCycleBio* detected more rhythmic transcripts than ECHO, this was mainly due to the identification of additional non-sinusoidal behaviours, which ECHO was not designed to detect.

### 3.3 Data modalities and sampling conditions influence rhythm type distribution

Reanalyzing previously published data from a circadian stud with high sampling frequency (Bignon *et al*. 2023), we investigated signal heterogeneity from different the transcriptome, proteome, and metabolome of murine renal tissue (Fig. 1G). Analysis of each dataset by *PyCycleBio* revealed different distributions of rhythm types between modalities. Interestingly, the metabolome had the greatest proportion of sinusoidal oscillations, in contrast to the transcriptome, where only 12.0% of oscillating transcripts were best modelled by a sinusoidal oscillation. The metabolome and proteome both exhibited a greater proportion of transient oscillations (13.9% and 14.2%, respectively) than the transcriptome (4.6%).

Utilizing transcriptomics data, in which mice were either fed *ad-libitum*, or calorie-restricted to two hours during the light or dark phases (Acosta-Rodríguez *et al*. 2022) (Fig. 1H). We observed shifts in the rhythmic content of the liver transcriptome following calorie-restriction, with sinusoidal oscillations being reduced from 47.1% in *ad-lib* fed mice to 29.0% in night-fed and 36.6%in day-fed mice. Additionally, the damping coefficient obtained with our analyses provided insights into regulatory behaviours of the observed rhythms, as 31.6% of rhythms observed in *ad-lib*-fed mice exhibited stable behaviour (not actively being up- or down-regulated during the sampled timeframe). Conversely, this was reduced to 31.2% of rhythms being stably expressed in night-fed animals, while in day-fed animals only 7.2% of rhythms were stable over the experimental time-course (Fig. 1I). This suggests stronger disruptions to diurnal behaviours can manifest as increased heterogeneity in the regulation and form of rhythmic transcriptome content, detectable via *PyCycleBio*, which should be considered in experimental design and physiological outcomes. We note that because functionality for differential-rhythmicity analysis is not yet included in *PyCycleBio*, some of these changes may be erroneous and reflect stochastic differences in signals. We hope to provide this functionality in future versions of *PyCycleBio.*

## 4 Discussion

*PyCycleBio* couples the amplitude-change coefficient used in ECHO (De Los Santos *et al*. 2020) with the multi-component models of Cosinor (Moškon 2020). We have validated the capacity of our approach to identify complex biological rhythms, demonstrating it to be as sensitive to sinusoidal rhythms as the current state-of-the-art (Fig. 1B, available as supplementary data at *Bioinformatics Advances* online) as well as vastly more performant (Fig. 1A, available as supplementary data at *Bioinformatics Advances* online). We note that the majority of rhythms detected by *PyCycleBio* are non-sinusoidal, evidencing the importance of considering complex dynamics (Fig. 1F). Additionally, the distribution of rhythmic models differed between data modalities. Metabolomics data appeared to have the highest sinusoidal content, while transcriptomics and proteomics data exhibited more complex dynamics (Fig. 1G). Post-transcriptional regulatory processes governing transcript and protein abundance could offer a possible explanation for this observation, as many distinct factors can contribute to the observed complex dynamics in these instances. We also note differences in the distribution of rhythmic behaviours between datasets (Fig. 1G and H), which may reflect distinct peripheral clock behaviours in different tissues, highlighting the interesting new lines of inquiry our approach facilitates. We envision *PyCycleBio* to be useful for the circadian and chronobiological fields, aiding our understanding of complex temporal phenomena and the underlying regulatory mechanisms. Additionally, we note that most temporal bioinformatics platforms have been developed and deployed using R. Developed in Python as open-source software*, PyCycleBio* can leverage, and be integrated alongside, computational platforms from other fields, such as data science and machine learning. We are thus confident *PyCycleBio* will enable chronobiologists to benefit from exciting developments in computational biology and artificial intelligence in the future.

## Supplementary Material

vbag018_Supplementary_Data

## Data Availability

Code and documentation are available via PyCycleBio and https://github.com/Glycocalex/PyCycleBio.
