# Supplementary material for: PyCycleBio: modelling non-sinusoidal-oscillator systems in temporal biology
Source: Bioinform Adv. 2026 Jan 22;6(1):vbag018. doi: 10.1093/bioadv/vbag018 (PMC12895064; doi:10.1093/bioadv/vbag018)
Supplement: vbag018_Supplementary_Data [file vbag018_supplementary_data.pdf]

A

Dataset (# of molecules)	PyCycleBio (mean of 10 reps)	ECHO (1 rep)	Times speed up
PMID32613882 *[SyntheÃc data]* (11,000)	190 sec (S.D. +/- 5sec)	4hrs, 27mins, 47 sec	85x
PMID29358041 (24,453)	128 sec (S.D. +/- 3sec)	2hrs, 33mins, 34 sec	72x
PMID34059820 (11,606)	135 sec (S.D. +/- 6sec)	2hrs, 41mins, 44 sec	72x
PMID34968386 (11,727)	132 sec (S.D. +/- 6sec)	2hrs, 40mins, 16 sec	73x
PMID35511946 (21,869)	159 sec (S.D. +/- 7sec)	4hrs, 53mins, 57 sec	111x

B

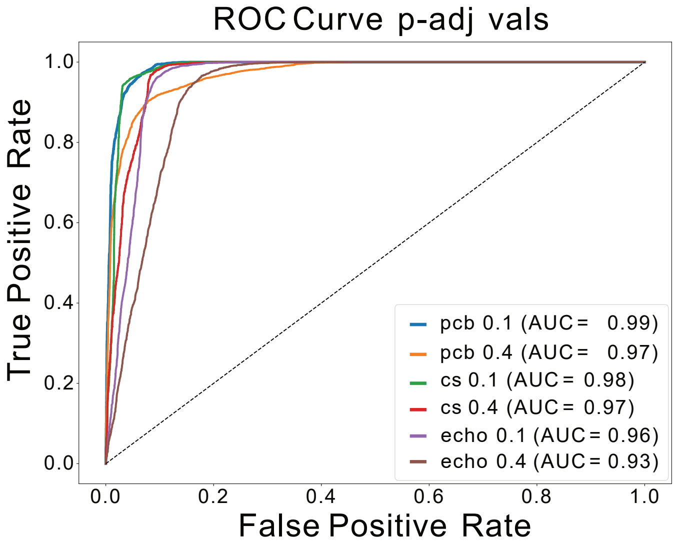

C

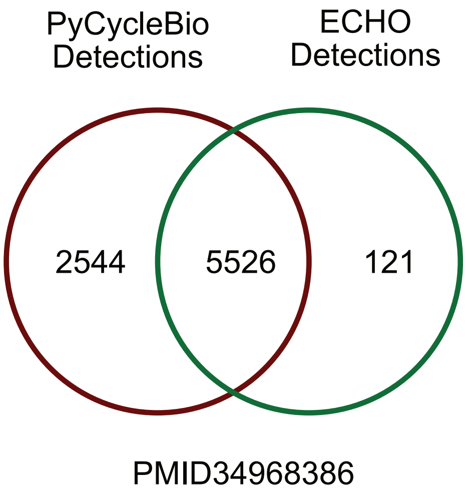

Supplementary figure 1:

(A) Receiver-operating-characteristic (ROC) curves for the accuracy of three platforms: *PyCycleBio* (pcb), *Cosinor* (cs), and *ECHO* (echo), when analysing synthetic sinusoidal data with a noise ratio of 0.1x or 0.4x total amplitude (Ness-Cohn, 2020). **(B)** A comparison of the time required to analyse circadian datasets, between *PyCycleBio* and *ECHO* (de Los Santos, *et al.* 2020). **(C)** A Venn-diagram showing the number of common and unique rhythmic transcripts detected by *PyCycleBio* and ECHO (using data from Bignon, *et al.* 2023).
